# Determination of Foetal Scalp Blood Sampling pH as an Indicator of Loss of Foetal Well-Being in Women Undergoing Caesarean Section

**DOI:** 10.3390/healthcare11050725

**Published:** 2023-03-02

**Authors:** Raquel Alarcón-Rodríguez, María Paz Martín-Álvarez, Jessica García-González, Mar Requena-Mullor, Mª Carmen Rodríguez-García, Ruirui Zheng, Raúl Romero-del Rey

**Affiliations:** 1 Department of Nursing, Physiotherapy, and Medicine, University of Almería, 04120 Almería, Spain; 2 Midwife in Poniente Hospital, 04700 Almería, Spain

**Keywords:** caesarean section, foetal distress, academia, hypoxia, APGAR score, blood gas analysis

## Abstract

Asphyxia during birth is one of the three leading causes of neonatal morbidity and mortality among newborns carried to term. The objective of this study was to evaluate the measurement of the foetal scalp blood pH as a measure of foetal status, evaluating: cord gases, meconium-stained fluid, APGAR score or the need for neonatal resuscitation in pregnant women undergoing caesarean sections. A cross-sectional study was carried out over a period of 5 years (2017–2021) at the Hospital de Poniente (southern Spain). A total of 127 pregnant women participated from whom a foetal scalp blood pH sample was taken and used to indicate the need for an urgent caesarean section. The results showed a correlation between the pH of the scalp blood and the pH of the umbilical cord artery, umbilical cord vein (Rho of Spearman arterial pH: 0.64, *p* < 0.001; Rho of Spearman venous pH: 0.58, *p* < 0.001) and the APGAR test one minute after delivery (Spearman’s Rho coefficient of 0.33, *p* < 0.01). These results suggest that the foetal scalp pH should not be considered a foolproof method to indicate an urgent caesarean section. Foetal scalp pH sampling can be used as a complementary test, in conjunction with cardiotocography, to indicate whether an emergency caesarean section is necessary due to loss of foetal well-being.

## 1. Introduction

The latest report on “Perinatal Care in Spain: Analysis of physical and human resources, hospital activity and quality of hospital services” states that, in 2018, there were 368,368 births in Spain [1], of which 21.8% were performed by caesarean section, about 7% more than the stipulated maximum set by the World Health Organization (10–15%) [2]. Birth asphyxia is one of the three leading causes of perinatal morbidity and mortality among newborns at term [3,4]. The most widely used indicators to evaluate perinatal asphyxia are the colour of the amniotic fluid, abnormal patterns in the foetal heartbeat [5], pH of the umbilical cord [6,7,8], pH of the scalp blood [9,10,11] and the APGAR test score immediately after birth [12,13,14], although it cannot currently be said that there is a strong association between these indicators and negative perinatal outcomes [15].

Monitoring foetal well-being throughout the birthing process is essential in obstetrics to reduce adverse perinatal outcomes [5,9]. Cardiotocography continues to be the most widespread tool for this monitoring; however, due to its low specificity, many obstetricians opt for a more objective test, such as the foetal scalp pH [5,16,17]. The intention of the foetal scalp blood pH test is to confirm if the foetus is at risk of intrapartum asphyxia and needs immediate delivery. In addition, this test attempts to override the false-positive foetal heart rate results suggesting traces of asphyxia, i.e., to identify false positives on cardiotocographic records [16,18]. In this regard, the widespread use of intrapartum cardiotocography has contributed to an increase in the rate of caesarean sections due to alleged foetal distress [3]. Foetal distress is the leading rationale for category-1 caesarean sections, which are associated with a low APGAR score, compared to non-category-1 caesarean sections [19,20]. Syed et al. (2020) report that low pH levels in the umbilical cord of babies born by caesarean section (due to foetal distress) are strongly correlated with a low APGAR score after delivery and higher rates of admission into the intensive care unit [3]. However, in recent years, due to a lack of randomised clinical trials, the factors that influence the foetal scalp pH, as well as limitations related to technical procedures, have left many researchers questioning if the determination of the pH of blood samples from the scalp is truly the “gold standard” among diagnostic tests to evaluate the foetal metabolic state [21,22,23,24]. Obstetricians should consider that foetal scalp blood values are not always reliable and may be false. For this reason, some authors recommend taking two blood samples routinely at each foetal scalp sampling attempt to confirm the foetal scalp blood pH [22].

A Cochrane review stated that there is insufficient evidence to link the use of foetal scalp pH testing and better long-term perinatal outcomes [25]. Furthermore, the use of foetal scalp pH testing elevates the number of caesarean sections and forceps deliveries, as well as serious foetal complications [26]. However, the rate of use of the foetal scalp pH testing varies widely among hospitals and countries, and the frequency of its use on women in labour depends largely on local practices [27]. Hilal et al. (2017) reported that the scalp blood pH test is not sensitive enough to reliably detect foetal acidosis and does not detect most true cases of acidosis. These authors recommend the foetal scalp pH to be taken as a complementary test, but every obstetrician should know foetal scalp pH sampling is an unreliable test for identifying foetal acidosis [10]. A recent study confirms that taking a scalp blood sample as a tool used in conjunction with cardiotocography may have a limited ability to predict neonatal acidemia, low APGAR scores and intensive care unit admission [9].

There are few studies in Spain that evaluate the usefulness of foetal scalp pH as an indicator of loss of foetal well-being in women who undergo a caesarean section. For these reasons, the aim of this study was to evaluate the measurement of the foetal scalp blood pH as a measure of foetal status, evaluating: cord gases, meconium-stained fluid, APGAR score or the need for neonatal resuscitation in women undergoing caesarean sections in the province of Almería (Spain).

## 2. Materials and Methods

### 2.1. Design

A cross-sectional study was conducted to evaluate the measurement of the foetal scalp blood pH as a measure of foetal status, evaluating: cord gases, meconium-stained fluid, APGAR score or need for neonatal resuscitation.

### 2.2. Study Population and Data Collection

The original study sample consisted of 510 adult pregnant women, who had undergone a caesarean section due to loss of foetal well-being after non-reactive cardiotocography, at the Hospital of Poniente in the province of Almería (southeast Spain) from 2017 to 2021. Out of the 510 possible participants, 383 were excluded for not meeting the inclusion criteria, leaving a final number of 127 women.

The inclusion criteria for this study were the following: pregnant women at over 39 weeks of gestation, 18 years of age or older, with a singleton pregnancy, who had undergone a caesarean section due to the risk of losing foetal well-being with non-reactive cardiotocography after taking the foetal scalp blood sample pH. The exclusion criteria were: pregnant women at less than 29 weeks of gestation, under 18 years of age and from whom coagulated or insufficient samples of both the pH of the scalp and the pH of the umbilical artery or vein had been taken. All the caesarean sections were performed in a maximum period of 30 min after the time the sample was taken and were performed on women whose newborns were carried to full term.

Cord blood samples were collected immediately after manual removal of the placenta. During caesarean section, the placenta is removed and the umbilical cord is clamped before cutting by the gynaecologist. Thus, the blood flow is stopped and the pH is maintained, ensuring the highest reliability of the sample. Subsequently, samples are taken from the umbilical cord area between the two clamps by the midwife. Gas syringes were used for sample collection, more specifically, one for arterial and one for venous samples, previously identified.

The time elapsed between foetal scalp blood sampling pH measurement and caesarean section was no more than 30 min.

The data were collected through the computer records of the Department of Obstetrics and Gynaecology, using the software Ariadna, where the digital medical records of all patients who are treated in this hospital are stored.

Sociodemographic variables of the mother and the newborn were collected including: weeks of gestation, age and nationality of pregnant women, marital status, cervical dilation, sex of the newborn and birth weight. Other data about the well-being of the newborn were also collected: scalp blood sample pH (threshold values pH > 7.21: normal and pH ≤ 7.20: acidemia [28]), umbilical artery pH (threshold values 7.20–7.34) [27], umbilical vein pH (threshold values 7.28–7.40) [27], APGAR score 1 min, 5 min and 10 min after birth (APGAR score < 7 was considered abnormal), neonatal resuscitation was classified as Type 0: no resuscitation required, Type I: aspiration of secretions, Type II: aspiration + oxygen and Type III: mask ventilation, and the colour of the amniotic fluid with meconium was classified as: clear, thin (+), medium (++) and thick (+++) [29].

The clinical protocol that was followed at the Hospital de Poniente (Almería, Spain) on the scalp pH value to perform an emergency caesarean section was the intrapartum foetal well-being control protocol of the Hospital Clinic de Barcelona (Spain) [27].

### 2.3. Instruments

To measure the scalp pH, samples from the scalp which consisted of a small amount of foetal blood were collected to assess intrapartum foetal well-being. Only the pH value was measured in the capillary samples. The gynaecologist was in charge of collecting the sample by inserting an amnioscope through the vagina to open the cervix and make a small incision in the foetal scalp. The small blood sample was collected in a heparin tube using capillary action. Once the sample was collected, it was analysed immediately in the hospital laboratory. To perform this technique, the patient must show no signs of sexually transmitted diseases and have a minimum dilation of 1 cm, and the amniotic sac must have ruptured.

### 2.4. Statistical Analysis

A database was created and statistical analysis was performed using SPSS version 25.0 (IBM^®^ Armonk, NY, USA). For the qualitative variables, frequencies with their corresponding percentages were calculated, and for the quantitative variables, the mean and standard deviation were calculated. For the comparison of means of quantitative variables, following a normality test (Kolmogorov–Smirnov Test), non-parametric tests were applied, as well as the Kruskal–Wallis test to compare independent variables. Quantitative variables were correlated using the Spearman correlation coefficient. A significance level of 95% was considered.

### 2.5. Ethical Considerations

The research study was approved by the Ethics Committee in Research from the University of Almería (EFM 136/2021). All the procedures were performed following the ethical standards of the Helsinki Declaration. The absolute anonymity and confidentiality of the data provided were guaranteed through the generation of a personal code.

## 3. Results

There was a total of 127 pregnant women included in the study. All the pregnant women had undergone a caesarean section due to the risk of losing intrauterine foetal well-being.

The pregnant women who participated in the study were 35 (SD 5.68) years old on average and the majority were married. The mean gestational age of the women was 39.85 (SD 1.29) weeks. 55.1% of the pregnant women were of Spanish nationality and 44.9% were of Moroccan nationality. 61.4% of the newborns were males, with a mean birth weight of 3.350 (SD 0.43) grams.

Cervical dilation of the pregnant women at the moment of taking a sample of pH from the scalp: 3.8 (0.96).

### 3.1. Risk Indicators for Loss of Foetal Well-Being

The mean pH of the scalp blood sample of the 127 pregnant women who underwent a caesarean section was 7.21 (SD 0.08). The mean arterial pH umbilical cord upon delivery was 7.19 (SD 0.08) and the pH of the umbilical vein was 7.23 (SD 0.07).

Regarding the APGAR test 1 min after delivery, the mean score of the newborns was 8.46 (SD 1.44), which increased on the subsequent APGAR tests performed 5 min and 10 min after delivery (Table 1).

After delivery, 63% of the newborns did not require any time of neonatal resuscitation, and only 15.7% needed Type III resuscitation. The colour of the amniotic fluid during the delivery was clear in 63% of the caesarean sections and 7.1% had thick (+++) amniotic fluid (Table 1).

### 3.2. Comparison of the pH of the Foetal Scalp Blood with Indicators of Foetal Well-Being during Delivery

No statistically significant differences were observed when comparing mean scores of the foetal scalp pH regarding neonatal resuscitation and the colour of the amniotic fluid (Table 2).

Table 3 shows the comparison amongst the type of neonatal resuscitation, the colour of the amniotic fluid and APGAR scores at 1 and 5 min of newborn life, regarding two groups: normal foetal scalp blood pH values (pH > 7.21) and acidemia (pH ≤ 7.20). Statistically significant differences were observed for the type of neonatal resuscitation, resulting in resuscitation Type III for 22.4% of newborns with pH ≤ 7.20 (acidemia) and 8.3% with pH > 7.21 (normal).

Regarding amniotic fluid colour, the amniotic fluid of newborns with thick meconium (+++) was significantly higher in babies with acidemia than in normal foetal scalp blood pH values (11.9% and 1.7%, respectively).

Furthermore, the APGAR test at minute 1 showed a significantly higher rate of abnormal results in babies with acidemia compared to those with normal foetal scalp blood pH values (19.4% and 8.3%, respectively).

No statistically significant differences were observed comparing APGAR tests at 5 min between groups, indicating that it was normal for all newborns in the study.

### 3.3. Correlation of the Foetal Scalp pH with Indicators of Foetal Well-Being in Newborns

The correlation between the foetal scalp pH and the arterial and venous pH of the umbilical cord after birth showed statistically significant results (Rho of Spearman arterial pH: 0.64, *p* < 0.001; Rho of Spearman venous pH: 0.58, *p* < 0.001).

Upon correlating the foetal scalp pH with the APGAR scores 1 min, 5 min and 10 min after birth, statistically significant results were only found in the APGAR test after 1 minute, with a Spearman’s Rho coefficient of 0.33 (*p* < 0.01).

## 4. Discussion

Although for many years, the foetal scalp pH has been considered the gold standard test for assessing foetal well-being during labour, it has never been duly validated, and the rationale for using it continues to be weak [21,22,23,24]. The study carried out by O’Brien and Murphy demonstrates the inconsistency of foetal blood pH results as evidence of foetal acidosis in labour, suggesting that foetal blood sampling should not be considered a foolproof test [30]. Currently, in modern obstetrics, there are studies advocating the use of foetal scalp stimulation, compared with foetal scalp pH sampling, although there is moderate to little evidence to support this practice [17,28,31]. Even FIGO recommended the use of foetal scalp stimulation in 2015, as it appears to have a predictive value similar to that of taking a scalp blood sample in the detection of foetal acidosis, and is also simpler and less invasive [32]. Our study found that the foetal scalp pH sample correlates significantly with the venous and arterial cord blood pH and the APGAR test results after one minute. The lowest foetal scalp pH values were obtained in newborns who required Type III neonatal resuscitation, and with heavily thick (+++) amniotic fluid, although these results were not statistically significant. The foetal scalp pH is considered an insufficient test to detect foetuses in acidosis, since in its realisation, there are numerous limitations that may contaminate the results of the test [22]. In this study, a significantly higher percentage of foetuses in acidosis were found to require Type III resuscitation compared to foetuses with normal pH values.

In addition, results of the amniotic fluid colour showed a significantly higher rate of newborns with thick meconium (+++) in babies with acidemia. Other authors support that contamination of the blood sample with amniotic fluid can lead to diverse results. On the one hand, if the fluid is clear, it would have an alkaline pH, which could be misleading for a diagnosis of foetal acidosis. On the other hand, if the fluid is stained with meconium, it can distort the pH result, leading to a false diagnosis of acidosis [24].

Not all samples showed similar results between foetal scalp pH and umbilical cord arterial pH, which was subsequently recorded. Different scalp blood samples from participants had a pH significantly higher or lower than that of the subsequent result of the arterial pH of the umbilical cord obtained after caesarean section. Given these outcomes, we agree with other studies that it would be necessary to take at least two samples of the foetal scalp pH to avoid unnecessary obstetric interventions due to false positive results [22,33]. We must not forget that this test gives us a one-time result at a specific point in time. In contrast, we also found a study that compared the foetal scalp pH with the umbilical artery pH, without finding significant differences, although it is true that a sample size of 82 cases may be considered insufficient [22].

The original purpose of the foetal scalp pH test was to serve as an additional test to the auscultation of an intermittent foetal heartbeat, with the aim of decreasing the number of caesarean sections. However, continuous cardiotocographic monitoring is currently used, and foetal scalp pH tests are conducted when the cardiotocographic record is not reassuring [16], giving rise to the opposite effect to its original purpose, and therefore increasing the number of caesarean sections and forceps deliveries.

In our study, foetal scalp pH testing was also used as a complement to continuous cardiotocographic monitoring. However, we do not believe that this increased the number of caesarean sections, given the fact that, out of 510 caesarean sections due to risk of loss of foetal well-being, only 127 were recommended based on the result of the foetal scalp pH. In this context, we found one randomised clinical trial comparing the use of continuous cardiotocographic and foetal scalp pH monitoring, with cardiotocographic recording alone and intermittent auscultation. In this clinical trial, the caesarean section rate was slightly reduced with continuous cardiotocographic recording and foetal scalp pH testing [34].

Other important aspects to bear in mind are the pain that the test may cause for pregnant women and the difficulty in collecting the sample for the obstetrician. A study found that women who received an epidural tolerated the test well, but those without an epidural considered it rather painful and uncomfortable. Obstetricians concluded that it is generally not difficult to take the sample, but there are situations that complicate it, such as <7 cm cervical dilation, whether the woman was experiencing pain, if she had a high body mass index and if the foetal head was still high [35].

In addition, the physiopathological foundation supporting the use of the foetal scalp blood sample pH could be erroneous, because it is known that hypoxia at the central level causes peripheral vasoconstriction to send blood to central organs in an attempt to compensate, leading to misleading peripheral acidosis [21]. In this regard, some studies have advocated that the measurement of lactates in the scalp blood is more reliable than pH, as well as a better assessment of the metabolic aspect [16,25,33,36]. Another study found discordant results when comparing scalp pH and lactates, especially when the cervix was fully dilated and when the amniotic fluid was meconium-stained [37]. This work only analysed pH, but it might be interesting to take other measurements such as lactate or base excess to provide more information on possible acidosis.

### Limitations and Strengths of This Study

This study has some limitations. On the one hand, we want to point out that there are many factors that can influence the one-time outcome of foetal scalp pH testing and that we were not able to take this into account in our study, either because they were not recorded on any official records or because sometimes these factors do not come to light until after delivery. For example, we were not able to confirm or deny the usefulness of foetal scalp stimulation compared to the use of scalp blood pH testing because, although it is true that we use it daily in our practice, we do not typically record it; in this sense, this could open the door to a future line of research that includes foetal scalp stimulation. Furthermore, another limitation would be that this is a retrospective single-centre trial. It would be advisable to carry out a prospective multicentre study or a randomised clinical trial in the future.

Among the strengths of this study, we would like to highlight that the development of this research challenges the use of certain clinical practises at some hospitals, which are not supported by consolidated scientific evidence. This study will serve as a basis for other studies to try to resolve the existing controversy on this subject.

## 5. Conclusions

In this study, foetal scalp pH correlated with the umbilical cord arterial pH, venous pH and APGAR test results 1 min after birth. These results suggest that the foetal scalp pH should not be considered foolproof to indicate the need for an urgent caesarean section. Foetal scalp pH sampling can be used as a complementary test, in conjunction with cardiotocography, to indicate whether an emergency caesarean section is required due to loss of foetal well-being.

The use of this test should be considered on a case-by-case basis, considering the other indicators of loss of foetal well-being, and should not use a single sample to designate the need for an urgent caesarean section.

## Figures and Tables

**Table 1:** Risk indicators of loss of foetal well-being.

Variables		Foetal Scalp pH		*p*-Value ^a^
		Normal (pH > 7.21) (*n* = 60)	Acidemia (pH ≤ 7.20) (*n* = 67)	
Type of neonatal resuscitation	Type 0	41 (68.3%)	39 (58.2%)	0.03
	Type I	14 (23.3%)	10 (14.9%)	
	Type II	0	3 (4.5%)	
	Type III	5 (8.3%)	14 (22.4%)	
Colour of the amniotic fluid	Clear	45 (75%)	40 (59.7%)	0.03
	Thin (+)	9 (15%)	7 (10.4%)	
	Medium (++)	5 (8.3%)	12 (17.9%)	
	Thick (+++)	1 (1.7%)	8 (11.9%)	
APGAR at minute 1	Normal	55 (91.7%)	54 (80.6%)	0.06
	Abnormal	5 (8.3%)	13 (19.4%)	
APGAR at 5 min	Normal	60 (100%)	67 (100%)	-
	Abnormal	0	0	

^a^ Result expressed as mean and standard deviation (SD).

**Table 2:** Comparison of the foetal scalp pH based on the type of neonatal resuscitation required and the colour of the amniotic fluid.

Variables		Mean	SD ^a^	*p*-Value ^b^
Type of neonatal resuscitation	Type 0	7.22	0.07	0.21
	Type I	7.23	0.08	
	Type II	7.17	0.01	
	Type III	7.18	0.10	
Colour of the amniotic fluid	Clear	7.21	0.08	0.46
	Thin (+)	7.25	0.08	
	Medium (++)	7.20	0.07	
	Thick (+++)	7.19	0.08	

^a^ SD = Standard deviation. *p*-value obtained with ^b^ Kruskal–Wallis test.

**Table 3:** Comparison of the normal and acidemia foetal scalp pH based on the type of neonatal resuscitation required, the colour of the amniotic fluid and the APGAR test.

Variables		Foetal Scalp pH		*p*-Value ^a^
		Normal (pH > 7.21) (*n* = 60)	Acidemia (pH ≤ 7.20) (*n* = 67)	
Type of neonatal resuscitation	Type 0	41 (68.3%)	39 (58.2%)	0.03
	Type I	14 (23.3%)	10 (14.9%)	
	Type II	0	3 (4.5%)	
	Type III	5 (8.3%)	14 (22.4%)	
Colour of the amniotic fluid	Clear	45 (75%)	40 (59.7%)	0.03
	Thin (+)	9 (15%)	7 (10.4%)	
	Medium (++)	5 (8.3%)	12 (17.9%)	
	Thick (+++)	1 (1.7%)	8 (11.9%)	
APGAR at minute 1	Normal	55 (91.7%)	54 (80.6%)	0.06
	Abnormal	5 (8.3%)	13 (19.4%)	
APGAR at 5 min	Normal	60 (100%)	67 (100%)	-
	Abnormal	0	0	

*p*-value obtained from ^a^ Chi-Square test.

## Data Availability

Data of this study are stored in an SPSS software project.
